# Differential selection and mutation between dsDNA and ssDNA phages shape the evolution of their genomic AT percentage

**DOI:** 10.1186/1471-2156-6-20

**Published:** 2005-04-11

**Authors:** Xuhua Xia, Kwok Yung Yuen

**Affiliations:** 1 Department of Biology, University of Ottawa, Ottawa, Ontario, K1N 6N5, Canada; 2 Department of Microbiology, University of Hong Kong, Hong Kong

## Abstract

**Background:**

Bacterial genomes differ dramatically in AT%. We have developed a model to show that the genomic AT% in rapidly replicating bacterial species can be used as an index of the availability of nucleotides A and T for DNA replication in cellular medium. This index is then used to (1) study the evolution and adaptation of the bacteriophage genomic AT% in response to the differential nucleotide availability of the host and (2) test the prediction that double-stranded DNA (dsDNA) phage should exhibit better adaptation than single-stranded DNA (ssDNA) phage because the rate of spontaneous deamination, which leads to C→T or C→U mutations depending on whether C is methylated or not, is about 100-fold greater in ssDNA than in dsDNA.

**Results:**

We retrieved 79 dsDNA phage and 27 ssDNA phage genomes together with their host genomic sequences. The dsDNA phages have their genomic AT% better adapted to the host genomic AT% than ssDNA phage. The poorer adaptation of the ssDNA phage can be partially accounted for by the C→T(U) mutations mediated by the spontaneous deamination. For ssDNA phage, the genomic A% is more strongly correlated with their host genomic AT% than the genomic T%.

**Conclusion:**

A significant fraction of variation in the genomic AT% in the dsDNA phage, and that in the genomic A% and T% of the ssDNA phage, can be explained by the difference in selection and mutation between them.

## Background

We first present a simple model of DNA replication to show that the genomic AT% of rapidly replicating bacterial species is indicative of the relative availability of nucleotides A and T in the bacterial cell. By using the genomic AT% of bacterial species as an index of AT availability, we study how bacteriophage would evolve in response to the differential AT availability in different bacterial hosts. We expect natural selection to favour the evolution of phage genomic AT% to take advantage of the differential AT availability in different hosts. In particular, given that the rate of spontaneous deamination, which results in C→T mutation (when the C is methylated or C→U mutations (when the C is not methylated), is 100-fold higher in single stranded DNA than in double-stranded DNA [1], we expect the adaptation of phage genomic AT% to their host cellular environment to be more disrupted by the C→T(U) mutations in single-stranded DNA (ssDNA) phage than in double-stranded DNA (dsDNA) phage.

Designate the amount of dATP, dCTP, dGTP and dTTP available for DNA replication as V_dA_, V_dC_, V_dG _and V_dT_, respectively. Note that these are abstract terms and may not correspond to the cellular concentration of dNTPs or rNTPs. Suppose a single-stranded DNA genome of length L is composed of A, C, G, and T with frequencies N_A_, N_C_, N_G _and N_T_, respectively (N_A _+ N_C _+ N_G _+ N_T _= L). The polymerization reaction is characterized as



where M_n_• stands for an elongating (or propagating in chemistry terminology) DNA strand with n monomer residues, M is the monomer, and k_p _is the propagating constant. According to the law of mass action, and assuming that k_p _is the same for adding any of the four nucleotides to the elongating chain, the elongation rate (r) during DNA replication can be modelled as



Bacterial species often need, and typically are selected, to replicate rapidly. For example, *E. coli *in unlimited culture conditions can replicate once every 20 minutes. It is therefore reasonable to assume natural selection to operate on increasing r for such organisms. According to equation (2), if V_dA _is the largest, then r is increased with increasing N_A _and decreasing N_G_, N_C _and N_T_, with the constraint of N_A _+ N_C _+ N_G _+ N_T _= L. The maximum r is achieved when N_A _= L and N_C _= N_G _= N_T _= 0. This means that, in order to maximize r with differential nucleotide availability, the genomic nucleotide usage should evolve to adapt to the availability of nucleotide availability by maximizing the usage of the nucleotide of the highest availability. Similar conclusions have also been derived elsewhere on optimization at the molecular level [2].

One should note that the model above does not consider the effect of differential depletion of the nucleotides. For example, consider that V_dA _is the largest among the four at the beginning of DNA replication. If a rapidly replicating genome is made entirely of A, then A will be differentially depleted leading to a reduced V_dA _which consequently may become smaller than V_dC_, V_dG _and V_dT_. This means that the replication of the remaining A-rich part of the genome would be slow, thus compromising the statement above that "The maximum r is achieved when N_A _= L and N_C _= N_G _= N_T _= 0". However, the qualitative conclusion that, if V_dA _is larger than V_dC_, V_dG _and V_dT_, then N_A _should be larger than N_G_, N_C _and N_T _remains correct.

When V_dC _= V_dG _= V_dA _= V_dT _= V, then equation (2) becomes:



so that r is independent of N_A_, N_C_, N_G_, and N_T_. This might be interpreted to mean that, with equal availability of the nucleotides for DNA replication, there is no selection on genomic nucleotide usage and genomic nucleotide frequencies can vary freely. However, the replication of a large, rapidly elongating and AT-rich genome may differentially reduce V_dC_, V_dG_, V_dA_, and V_dT_. For example, rapid replication of a large AT-rich genome will reduce V_dA _and V_dT _and increase the time for adding the remaining A and T to the elongation chain. Thus, even with V_dC _= V_dG _= V_dA _= V_dT _= V at the beginning of the replication, we would still expect the genomic AT% to be near 50% instead of fluctuating to extreme values.

For a double-stranded genome where N_A _= N_T _= N_AT _and N_C _= N_G _= N_CG_, equation (2) becomes



If V_dA_*V_dT _>> V_dC_*V_dG_, then increasing N_AT _in the genome will increase r, with the maximum r achieved when N_AT _= L and N_CG _= 0, i.e., the genome should evolve towards AT-richness. Again, this assumes no differential depletion of A and T and should be interpreted qualitatively to mean that, with V_dA_*V_dT _>> V_dC_*V_dG_, we should have N_AT _> N_CG_.

If V_dA_*V_dT _= V_dC_*V_dG_, then r becomes independent of N_AT _and N_GC_. However, this again does not necessarily mean that there is no selection to constrain genomic AT% and that genomic AT% can consequently vary freely. As we have argued before, a large, rapidly replicating and AT-rich genome will differentially reduce nucleotides A and T and lead to V_dA_*V_dT _<< V_dC_*V_dG _which is unfavourable for replicating an AT-rich genome. Thus, with V_dA_*V_dT _= V_dC_*V_dG_, we expect the genomic AT% to be near 50% instead of fluctuating to extreme values.

In summary, we expect an extremely GC-rich bacterial genome to indicate high V_dC_*V_dG_, an extremely AT-rich bacterial genome to indicate high V_dA_*V_dT_, and a bacterial genome with GC% = 50% to indicate (V_dA_*V_dT_) ≈ (V_dC_*V_dG_).

Based on the reasoning above, we may infer that different genomic AT% values in different bacterial species indicate different AT availability in the cells of these bacterial species. By using the genomic AT% of bacterial species as an index of AT availability, we now study how bacteriophage genomic GC% evolve in response to different nucleotide availability in different hosts.

Assuming that it is beneficial for the phage to replicate its genome rapidly, we can make two testable predictions. First, a phage genome should evolve to become AT-rich in a host with a high genomic AT% (indicating V_dA_*V_dT _>> V_dC_*V_dG _in its cell), and GC-rich in a host with a low genomic AT% (indicating V_dA_*V_dT _<< V_dC_*V_dG _in its cell). This will lead to a positive correlation between the phage genomic AT% and the host genomic AT%. Such a correlation has in fact been known for a long time [3]. Second, because the rate of spontaneous deamination, which leads to C→T or C→U mutations depending on whether C is methylated or not, is about 100-fold higher in the ssDNA than in dsDNA [1], we expect such mutations to reduced the effectiveness of natural selection to optimize the genomic AT% of the ssDNA phage in response to their host genomic AT%. In particular, with low host AT availability, natural selection should favour the reduction of the phage genomic AT%, but the C→T(U) mutation mediated by the spontaneous deamination in the ssDNA phage would counteract against natural selection and increase the genomic AT% of the ssDNA phage. In addition, because genomic A% and T% can evolve independently in ssDNA phage, we can specifically predict an increase in the genomic T% in ssDNA phage without an associated increase in the genomic A%. We will test these predictions.

## Results

**Figure 1:**
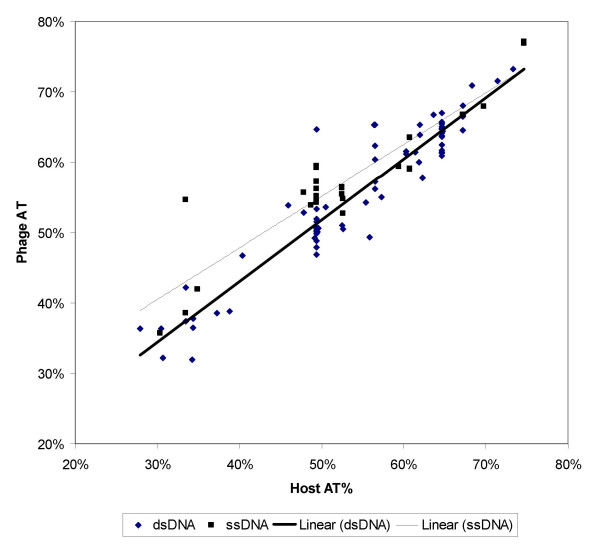
**Relationship between the phage genomic AT% and the host genomic AT%. **Data points for ssDNA and dsDNA phages are plotted separately with their respective linear regression lines.

The positive relationship between the phage genomic AT% and their host genomic AT% is shown separately for the dsDNA and ssDNA phages (Fig. 1). Such a positive relationship itself is trivial because the relationship has been known for nearly 40 years [3]. However, the difference between the dsDNA and ssDNA phages is scientifically significant. The regression line for the ssDNA phage has a higher intercept and a lower slope than that for the dsDNA phage (Fig. 1).

We can employ the general linear model (GLM) to test the statistical difference of the two regression lines:



where PhageAT and HostAT are AT% of the phage and host genomes, respectively, and PhageType is of two categories (i.e., dsDNA and ssDNA) coded by a binary dummy variable with 0 for ssDNA and 1 for dsDNA. If B_2 _and B_3 _are not significantly greater than 0, then there is no significant difference between the two regression lines.

The parameters of the general model in equation (5) can be evaluated by the GLM procedure in SAS [4], which uses the method of least-squares to fit general linear models. The resulting B_2 _is -10.156 which is statistically significant (t = 2.83, p = 0.0028). Similarly, B_3 _= 0.135, t = 2.04, p = 0.0221. The other parameters are B_0 _= 18.503 (t = 6.08, p < 0.0001), and B_1 _= 0.734 (t = 12.93, p < 0.0001)

Given the evaluated parameters in equation (5), the relationships between PhageAT and HostAT for dsDNA and ssDNA phages are



The increased intercept and decreased slope in the ssDNA phage relative to the dsDNA phage is easy to interpret in light of the finding that the rate of spontaneous deamination, which increases the C→T(U) mutation rate, is about 100-fold higher in ssDNA than in dsDNA [1]. This spontaneous deamination features prominently among all other factors contributing to the degradation of DNA [5]. When host genomic AT% is low (the left extreme of Fig. 1), indicating low availability of nucleotides A and T in the cellular medium according to equations (2) and (4), natural selection should cause the phage genome to reduce its AT%, but the C→T(U) mutation mediated by the high rate of spontaneous deamination in the ssDNA phage goes against natural selection and increases phage genomic AT%. In other words, the C→T(U) mutations reduce the effect of the natural selection to push the phage genomic AT% downwards. This would raise the intercept and decrease the slope of the regression for the ssDNA phage relative to the regression line for the dsDNA phage.

Note that the C→T(U) mutations act in the same direction as the natural selection when the host genomic AT% is high, indicating high availability of nucleotides A and T in the cellular medium according to equations (2) and (4). In this case, natural selection should favour phage genomes to become AT-rich, and the C→T(U) mutation mediated by the high rate of spontaneous deamination in the ssDNA phage also increases phage AT%, i.e., the two act in the same direction. Such an interpretation is consistent with the right side of Fig. 1 in which few points are below the regression line and with little scatter above and below the regression line, especially when the host genomic AT% is extremely high.

**Figure 2:**
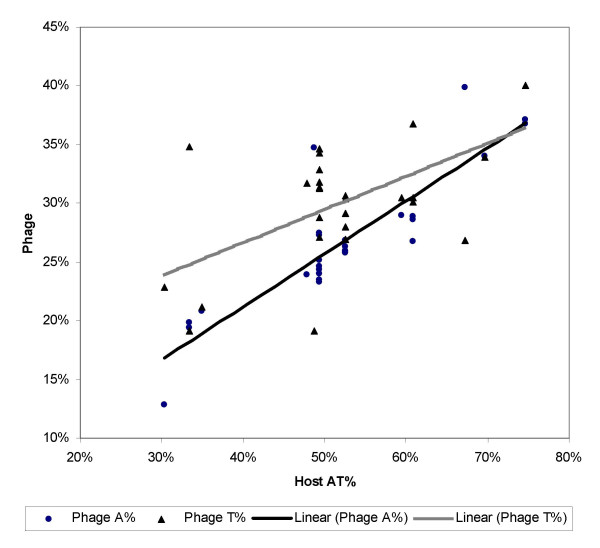
**The genomic A% and T% of the ssDNA phage plotted against their host genomic AT%. **The regression lines are separately fitted for the phage genomic A% and T%, respectively

To further substantiate this interpretation, we can test whether the increased intercept and decreased slope for the regression line of the ssDNA phage in Fig. 1 is really due to an increase in the genomic T% instead of the genomic AT%. This can be done because A and T do not need to be equal to each other in number for ssDNA. We expect an increased genomic T% but not genomic A% in the ssDNA phage. Such an inference is consistent with plotting the genomic A% and T% separately for the ssDNA phage against the host AT% (Fig. 2).

We can test the statistical significance of the difference between the two regression lines in Fig. 2 by using the general linear model similar to the approach in equations (5) and (6). The regression line for the genomic T% has a significantly increased intercept (P = 0.0068, one-tailed test) and decreased slope (P = 0.0323, one-tailed test). Also, the relationship between the phage genomic A% and the host genomic AT% is stronger than that between the phage genomic T% and the host genomic AT%, with the Pearson correlation coefficient being 0.87857 for the former and 0.60249 for the latter.

The results above corroborate our interpretation that C→T(U) mutations contribute significantly to the relationship in nucleotide frequency distribution between the phage genome and the host genome. In particular, the increased intercept and decreased slope for ssDNA phage in Fig. 1 can be largely attributed to the C→T(U) mutations mediated by the spontaneous deamination.

The pattern in Fig. 2, however, can have an alternative explanation. First, it is important to note that the host genomic AT% is only indicative of V_dA_*V_dT_. If V_dT _is similar in all hosts, but V_dA _differs substantially among hosts, then V_dA_*V_dT _will also differ substantially and phage genomic AT% will consequently be selected to adapt to the host environment of different V_dA_*V_dT_. However, for ssDNA phages in such a scenario with the hosts differing much in V_dA _but little in V_dT_, only the genomic A%, but not the genomic T%, of the ssDNA phages will show a good correlation with the host genomic AT%. This is also consistent with the pattern in Fig. 2.

## Discussion

Mutation and selection are the two sculptors of nature, but the effect of mutation on the evolution of genomes and proteins is only recently appreciated [6-9], notably after the pioneering work of Sueoka [10]. The C→T(U) mutations mediated by spontaneous deamination [1,5,11,12], in particular, have been invoked to explain the strand-asymmetry in nucleotide frequency distribution in vertebrate mitochondrial genomes [13-15], in the bacterial genomes [16-18], and in coding sequences [19-22]. In this paper, we have shown how the C→T(U) mutations can operate together with selection to shape the genomic AT% of dsDNA phage and the genomic A% and T% in ssDNA phage.

Previous studies have shown that spontaneous mutation appears to be AT-biased in different genomes and genetic backgrounds [23-26], and the evidence is convincing based on the comparison between functional genes and their pseudogene counterparts [25,26]. However, mutation alone is often insufficient to explain the observed genetic variation.

Two different kinds of AT-richness have been documented for mitochondrial genomes alone demanding two different explanations [2]. The first kind is represented by (1) the insect mitochondrial genomes where most codons end with A and T and (2) the mammalian mitochondrial D-loop which is not transcribed and very AT-rich. Both the D-loop and the third codon position of protein-coding genes evolve rapidly. In the insect mitochondrial genomes, the number of A-ending codons roughly equals the number of T-ending codons. In the D-loop, the number of A and T are distributed roughly equally in the two strands. This first kind of AT-richness was attributed to AT-biased mutation [2]. The second kind of AT-richness is represented by the coding sequences in vertebrate mitochondrial genomes, where most codons in four-fold degenerate codon families end with A but few end with T. This cannot be explained by the mutation hypothesis invoking AT-biased mutation because such mutations would lead to roughly equal number of A-ending and T-ending codons in four-fold degenerate codon families.

The large number of A-ending codons with few T-ending codons in mammalian mitochondrial genomes prompted the proposal of the transcription hypothesis of codon usage [2], based on the observation that cellular concentration of ATP is much higher than that of the other three rNTPs [Table 2.1 in [27-29], pp. 4–5]. For example, in the exponentially proliferating chick embryo fibroblasts in culture, the concentration of rATP, rCTP, rGTP and rUTP, in units of (moles × 10^-12 ^per 10^6 ^cells), is 1890, 53, 190, and 130, respectively, in 2-hour culture, and 2390, 73, 220, and 180, respectively, in 12-hour culture. The transcription hypothesis of codon usage states that, with the high availability of rATP and relatively low availability of the other three rNTPs, the transcription efficiency can be increased by maximizing the use of A in the third codon position of protein-coding genes.

The variation of the genomic AT% in the dsDNA phage and the genomic A% and T% in the ssDNA phage in our study cannot be explained by the C→T(U) mutations alone, and we believe that the correlations shown in Figs. 1,2 are mainly the work of natural selection favouring the AT-rich phage in AT-rich hosts and AT-poor phage in AT-poor hosts. The data from ssDNA phage helped us to conclude that it is the C→T(U) mutations, instead of AT-biased mutations, are mainly responsible for the difference between the ssDNA and dsDNA phages we observe in Figs. 1,2. The results in this paper corroborate our previous finding [15] that spontaneous deamination has profound effect on the strand-biased nucleotide and codon frequency distributions and on the codon-anticodon adaptation in another kind of intracellular genomes, i.e., the vertebrate mitochondrial genomes.

## Conclusion

The phage genomic AT% has evolved in response to the availability of A and T in their host cell. In particular, the difference in the relationship between the ssDNA phage and dsDNA phage, can be partially explained by the difference in (1) selection operating to maximize the rate of DNA replication and (2) the C→T(U) mutation mediated by the high rate of spontaneous mutations in the ssDNA phage.

## Methods

We have downloaded complete bacteriophage genomes for 79 dsDNA phages and 27 ssDNA phages in GenBank format from NCBI [30]. Nucleotide frequencies and AT% for each viral genome was calculated from the GenBank sequence files. The host species of each viral genome is taken from the "specific_host" entry in the FEATURES table of the phage sequence file [see Additional file 1], and the genomic AT% for the host species is calculated as follows.

### The genomic AT% for bacterial hosts with a genomic sequence

If the bacterial host is a particular strain of a particular species, and if the genomic sequence of that particular strain is available, then the genomic AT% was calculated from the genomic sequence. If the "specific_host" does not include a specification of the strain, and if several strains of the same bacterial species have complete genomic sequences, then the genomic AT% of the host species is the weighted average of these genomic sequences. This group consists of 82 cases out of the total of 118 phage-host pairs.

### The genomic AT% for bacterial hosts without a genomic sequence

Among the total of 118 phage-host pairs, 36 cases have the bacterial host without a completely sequenced genome. The genomic AT% of these bacterial hosts is estimated from sequences retrieved from GenBank as follows. First, we perform an ENTREZ search of the host species name with the limit of sequences set to "Genomic DNA/RNA" and with the exclusion of ESTs, STSs, GSSs, and patented sequences. Second, we deleted all plasmid sequences in the retrieved files. From the remaining sequences, one might then compute AT% from the retrieved sequences as the weighted average:



where n is the number of retrieved sequences for the host species, N_A + Ti _is the number of A and T for the i^th ^sequence, and L_i _is the length of the i^th ^sequence.

One problem with this calculation is that some genes from the same bacterial host have been sequenced and deposited multiple times and the resulting P_A + T _would tend to be over-represented by those genes present in multiple copies. For example, among 292 DNA sequences deposited in GenBank for *Acinetobacter sp.*, 152 are rRNA sequences (mostly 16S rRNA sequences), and all sequences deposited in GenBank for *Roseobacter sp. *are rRNA sequences. For this reason, we have first identified these genes by BLASTing [31] the sequences against each other with E-value = 0.0001 and calculated AT% by representing each set of multiply sequenced genes by the consensus sequence.

Note that this treatment may still suffer from biases. For example, DNA sequences of extreme GC% values (e.g., extremely GC-poor ones) may be more difficult to obtain than those with middle-range GC% values and are consequently underrepresented in GenBank. For this reason, we have also chosen the longest DNA sequence for each bacterial host as a genomic sample. The two sets of AT% values, with one set calculated as the weighted average and the other from the longest sequence in GenBank, are highly correlated (r = 0.975). The conclusions reached in this paper remains the same regardless of which set of the host AT% values we use. We present here only numerical results from representing each of these 36 bacterial hosts without a genomic sequence by its longest GenBank sequence. We have also performed an analysis by using only completely sequenced genomes. The sequence retrieval and analysis were carried out by using DAMBE [32,33].

In a comparative study, one should not treat each bacteriophage or bacterial host as providing an independent data point because of shared ancestry. For illustration, consider an extreme case with two clades of bacterial hosts, with each clade of species having close phylogenetic relationship and each infected by a clade of closely related phage species. We would essentially have only two data points when studying the relationship in AT% between the bacteriophage and the host, no matter how many species of bacterial hosts or bacteriophage species we have in each clade. Ideally, one should perform a phylogeny-based comparison as was done before [e.g., [34,35]]. Unfortunately, it is difficult to build a phage tree because, while a tree can be reconstructed for the bacterial species by using universally shared genes such as rRNA sequences, there is no such shared sequence among bacteriophage species. However, the phage genomic AT% appears to show little phylogenetic inertia. For example, for pairs of bacteriophage species (say A and B) in our study that share homology in protein-coding genes (indicating phylogenetic affiliation), the similarity in AT% between A and B is, in general, smaller than the similarity in AT% between each of them and their respective hosts (i.e., between A and the host of A and between B and the host of B). For this reason, we have adopted a technically undesirable, but approximately true, assumption of little phylogenetic inertia in bacteriophage AT%, with a caution for the reader that the probabilities associated with significance tests may not be exact. This assumption is somewhat justifiable based on the recent documentation of the lack of phylogenetic inertia in GC% (or AT%) of bacterial genomes [6]. If there is little phylogenetic inertia in bacterial genomes, then there should be even less phylogenetic inertia in the phage genomes because the latter evolve much faster than the former.

## Authors' contributions

XX and KYY conceived the project. XX obtained funding, carried out the project and drafted the paper. KYY participated in revising four earlier versions of the manuscript.

## Supplementary Material

Additional File 1 **List of bacteriophage and their hosts. **Also included are the genomic AT% of the host species and the phage nucleotide frequencies, in html format. Click here for file

## References

[B1] Frederico LA, Kunkel TA, Shaw BR (1990). A sensitive genetic assay for the detection of cytosine deamination: determination of rate constants and the activation energy. Biochemistry.

[B2] Xia X (1996). Maximizing transcription efficiency causes codon usage bias.. Genetics.

[B3] Gibbs A, Primrose S (1976). A correlation between the genome compositions of bacteriophages and their hosts.. Intervirology.

[B4] SAS Institute Inc. (1989). SAS/STAT User's guide. Version 6, Volume1..

[B5] Lindahl T (1993). Instability and decay of the primary structure of DNA.. Nature.

[B6] Gu X, Hewett-Emmett D, Li WH (1998). Directional mutational pressure affects the amino acid composition and hydrophobicity of proteins in bacteria. Genetica.

[B7] Hickey DA, Singer GA (2004). Genomic and proteomic adaptations to growth at high temperature. Genome Biol.

[B8] Wang HC, Singer GA, Hickey DA (2004). Mutational bias affects protein evolution in flowering plants. Mol Biol Evol.

[B9] Lobry JR (2004). Life history traits and genome structure: aerobiosis and G+C content in bacteria.. Lecture Notes in Computer Science.

[B10] Sueoka N (1961). Correlation bewteen base composition of deoxyribonucleic acid and amino acid composition of proteins.. Proceedings of the National Academy of Sciences, USA.

[B11] Frederico LA, Kunkel TA, Shaw BR (1993). Cytosine deamination in mismatched base pairs. Biochemistry.

[B12] Sancar A, Sancar GB (1988). DNA repair enzymes. Annu Rev Biochem.

[B13] Reyes A, Gissi C, Pesole G, Saccone C (1998). Asymmetrical directional mutation pressure in the mitochondrial genome of mammals. Mol Biol Evol.

[B14] Tanaka M, Ozawa T (1994). Strand asymmetry in human mitochondrial DNA mutations. Genomics.

[B15] Xia X (2005). Mutation and Selection on the Anticodon of tRNA Genes in Vertebrate Mitochondrial Genomes.. Gene.

[B16] McInerney JO (1998). Replicational and transcriptional selection on codon usage in Borrelia burgdorferi. Proc Natl Acad Sci U S A.

[B17] Lobry JR (1996). Asymmetric substitution patterns in the two DNA strands of bacteria. Mol Biol Evol.

[B18] Lobry JR, Sueoka N (2002). Asymmetric directional mutation pressures in bacteria. Genome Biol.

[B19] Beletskii A, Bhagwat AS (1996). Transcription-induced mutations: increase in C to T mutations in the nontranscribed strand during transcription in Escherichia coli. Proceedings of the National Academy of Sciences of the United States of America.

[B20] Beletskii A, Bhagwat AS (1998). Correlation between transcription and C to T mutations in the non-transcribed DNA strand. Biological Chemistry.

[B21] Beletskii A, Grigoriev A, Joyce S, Bhagwat AS (2000). Mutations induced by bacteriophage T7 RNA polymerase and their effects on the composition of the T7 genome. Journal of Molecular Biology.

[B22] Beletskii A, Bhagwat AS (2001). Transcription-induced cytosine-to-thymine mutations are not dependent on sequence context of the target cytosine. Journal of Bacteriology.

[B23] Marcelino LA, Andre PC, Khrapko K, Coller HA, Griffith J, Thilly WG (1998). Chemically induced mutations in mitochondrial DNA of human cells: mutational spectrum of N-methyl-N'-nitro-N-nitrosoguanidine. Cancer Res.

[B24] Wang RF, Campbell W, Cao WW, Summage C, Steele RS, Cerniglia CE (1996). Detection of Pasteurella pneumotropica in laboratory mice and rats by polymerase chain reaction. Lab Anim Sci.

[B25] Gojobori T, Li WH, Graur D (1982). Patterns of nucleotide substitution in pseudogenes and functional genes. J Mol Evol.

[B26] Li WH, Wu CI, Luo CC (1984). Nonrandomness of point mutation as reflected in nucleotide substitutions in pseudogenes and its evolutionary implications. Journal of Molecular Evolution.

[B27] Kornberg A, Baker TA (1992). DNA replication..

[B28] Colby C, Edlin G (1970). Nucleotide pool levels in growing, inhibited, and transformed chick fibroblast cells.. Biochemistry.

[B29] Bridger WA, Henderson JF (1983). Cell ATP.

[B30] NCBI NCBI Viral genomes. http://www.ncbi.nlm.nih.gov/genomes/VIRUSES/viruses.html.

[B31] Altschul SF, Gish W, Miller W, Myers EW, Lipman DJ (1990). Basic local alignment search tool.. Journal of Molecular Biology.

[B32] Xia X (2001). Data analysis in molecular biology and evolution..

[B33] Xia X, Xie Z (2001). DAMBE: Software package for data analysis in molecular biology and evolution. J Hered.

[B34] Xia X, Hafner MS, Sudman PD (1996). On transition bias in mitochondrial genes of pocket gophers.. Journal of Molecular Evolution.

[B35] Xia X (1998). The rate heterogeneity of nonsynonymous substitutions in mammalian mitochondrial genes.. Molecular Biology and Evolution.

